# Threshold effect of atherogenic index of plasma on type 2 diabetes mellitus and modification by uric acid in normal-weight adults with hypertension

**DOI:** 10.3389/fendo.2024.1495340

**Published:** 2024-11-27

**Authors:** Yu Tao, Tao Wang, Wei Zhou, Lingjuan Zhu, Chao Yu, Huihui Bao, Juxiang Li, Xiaoshu Cheng

**Affiliations:** ^1^ Department of Cardiovascular Medicine, the Second Affiliated Hospital of Nanchang University, Nanchang, Jiangxi, China; ^2^ Jiangxi Provincial Cardiovascular Disease Clinical Medical Research Center, Nanchang, Jiangxi, China; ^3^ Center for Prevention and Treatment of Cardiovascular Diseases, the Second Affiliated Hospital of Nanchang University, Nanchang, Jiangxi, China

**Keywords:** threshold effect, atherogenic index of plasma, uric acid modification, hypertension, type 2 diabetes mellitus

## Abstract

**Background:**

The association between atherogenic index of plasma (AIP) and type 2 diabetes mellitus (T2DM) in normal-weight individuals with hypertension remains unclear. This study seeks to elucidate this relationship in normal-weight adults with hypertension.

**Methods:**

This cross-sectional study included 8,258 normal-weight adults with hypertension from the China Hypertension Registry Study. The AIP was calculated as log10 (triglycerides/high-density lipoprotein cholesterol). The multivariate logistic regression, generalized additive model, smooth fitting curve, sensitivity analyses, two-part logistic regression, and subgroup analyses were conducted to detect the correlation between AIP and T2DM.

**Results:**

The mean age of the study population was 64.89 ± 8.97 years, with an overall prevalence of T2DM of 15.55%. Multivariate logistic regression analyses indicated that there was a positive and independent relationship between AIP and T2DM (OR: 3.73; 95% CI: 2.82, 4.94). Threshold effect analysis identified a J-shaped association between AIP and T2DM, with an inflection point at 0. Additionally, an interaction between hyperuricemia and AIP was observed (*P* for interaction = 0.034).

**Conclusions:**

In normal-weight adults with hypertension, there was a J-shaped association between AIP and T2DM, with an inflection point at 0. the correlation between AIP and T2DM was more pronounced in individuals with hyperuricemia compared to those with normal uric acid.

## 1 Background

As a prevalent chronic disease, diabetes has emerged as a substantial obstacle to global public health and healthcare expenditure. The Global Burden of Disease Study 2021 predicted that the global prevalence of diabetes would affect 529 million individuals in 2021, reflecting a global age-standardized total diabetes prevalence of 6.1%. Projections for 2050 estimated that more than 1.31 billion would suffer from diabetes, with 43.6% of nations exhibiting an age-standardized prevalence rate exceeding 10%. Type 2 diabetes mellitus (T2DM), which represents the majority of diabetes, constitutes 96% of all diabetes cases (1). The incidence of diabetes in China is escalating annually, as evidenced by the International Diabetes Federation (IDF) Diabetes Atlas, which indicates that by 2021, approximately 140 million individuals in China had been diagnosed with diabetes (2).

Hypertension frequently manifests concomitantly with diabetes, and studies have reported that over two-thirds of hypertensive individuals also present with diabetes (3, 4). In addition, hypertension can further complicate the medical management of diabetic patients by amplifying the severity of their microvascular damage (5). T2DM is frequently characterized by a gradual onset and an asymptomatic disease course; however, it may manifest as diabetic ketoacidosis (DKA) under conditions of physiological stress or improper medication management (6). In clinical settings, the utilization of Hemoglobin A1c (HbA1c) and Oral Glucose Tolerance Test (OGTT) is not routinely employed in general health examinations, primarily due to cost and accessibility constraints. Consequently, a significant number of diabetic patients may remain undiagnosed for extended periods, potentially leading to the development of associated diabetic complications. Statistical data reveals that approximately 50% of individuals suffering from diabetes globally remain undiagnosed (7). Consequently, the discovery of a straightforward and efficient index for identifying high-risk individuals susceptible to T2DM within the hypertension population is of significant clinical importance. This is particularly relevant in the context of early clinical intervention, as it can aid in preventing T2DM and its associated complications.

Dyslipidemia is highly prevalent in patients diagnosed with T2DM. A research study conducted in a Jordanian population revealed an 95.4% prevalence rate of dyslipidemia among individuals with T2DM (8). Diabetic dyslipidemia are characterized by hypertriglyceridemia, an increased proportion of small-dense low density lipoprotein (sdLDL) particles, elevated levels of remnant particles and decreased levels of high-density lipoprotein cholesterol (HDL-C) (9). Several studies have demonstrated that dyslipidemia is an independent risk factor for insulin resistance (IR) and T2DM (10–12). Furthermore, Longo et al. found that double mutation in genes linked to heterozygous familial hypercholesterolemia was independent predictors of T2DM from a genetic perspective. This association may be attributed to the multi-level interaction between the insulin receptor and the LDL receptor (13). Recently, a new lipid parameter, atherogenic index of plasma (AIP), has been proposed, which is converted from the logarithm of the ratio between the triglyceride (TG) and HDL-C. It was found to be negatively correlated with sdLDL, a subtype of low-density lipoprotein cholesterol (LDL-C), which possesses substantially enhanced atherogenic potential than other subtypes (14). Previous studies have revealed that AIP can be regarded as a powerful and reliable predictor of hypertension, atherosclerosis, coronary artery disease, nephropathy, nonalcoholic fatty liver disease and cardiovascular mortality (15–20). Furthermore, mounting researches have demonstrated the utility of AIP in evaluating the susceptibility to insulin resistance and the onset of T2DM. Individuals exhibiting elevated AIP levels are more predisposed to the development of insulin resistance and T2DM (21, 22). Notably, there are mounting evidences that substantiates a more robust correlation between AIP and T2DM in contrast to conventional lipid profiles (23, 24). Nevertheless, the aforementioned research primarily focused on non-hypertensive populations. Consequently, the relationship between AIP and T2DM remains ambiguous among populations with hypertension, who exhibit a heightened risk of cardiovascular disease. To address this research gap, this study endeavored to investigate the association between AIP and T2DM within normal-weight adults with hypertension. Additionally, the study sought to explore potential factors that could modify this relationship.

## 2 Methods

### 2.1 Participants

The sample for the current research is derived from the China Hypertension Registry Study (Registration number: ChiCTR1800017274), an ongoing observational registration study conducted in Wuyuan, Jiangxi province of China during the period from March 2018 to August 2018. The study protocols have been previously described (25). The study aimed to investigate the incidence of hypertension, the condition of hypertension control, and potential risk factors affecting the prognosis of hypertension in a real-world setting. The study’s inclusion criteria comprised of individuals aged 18 years or older who had hypertension. Hypertension was defined as having a sitting systolic blood pressure (SBP) of ≥ 140 mmHg or a diastolic blood pressure (DBP) of ≥ 90 mmHg, self-reported history of hypertension, or current use of antihypertensive medication (26). The exclusion criteria comprised of the following: 1) individuals with psychological disorders or nervous system impairments that hindered their ability to comprehend informed consent; 2) participants who were unable to comply with the study protocol’s long-term follow-up requirements or had plans to relocate in the near future; or 3) individuals deemed unsuitable for inclusion or long-term follow-up by study physicians. The Ethics Committee of the Institute of Biomedicine, Anhui Medical University (NO. CH1059) and the Second Affiliated Hospital of Nanchang University (NO. 2018019) granted approval for the study protocol. Informed consent was obtained from all participants who were recruited for this study.

A hypertension cohort was formed by consecutively enrolling 14,268 adults. Subsequently, 34 non-hypertensive individuals, 5,976 individuals with an abnormal weight according to the World Health Organization (WHO) standards (BMI < 18.5, *n* = 975; BMI ≥ 25, *n* = 4,720), and 261 individuals taking lipid-lowering drugs were excluded. Ultimately, 8,258 normal-weight adults with hypertension were analyzed (**Supplementary Figure S1**).

### 2.2 Data collection

The study obtained demographic characteristics through questionnaire surveys administered by uniformly trained staff. The questionnaire content encompassed sex, age, smoking history, drinking history, medical history, and medication information. Anthropometric parameters included height, weight, waist circumference (WC), SBP, and DBP. Height and weight were measured without shoes, while WC was assessed at the end of expiration in an erect position using a cloth tape. The blood pressure of all participants was assessed using a consistent electronic sphygmomanometer (HBP-1300; Omron, Japan) while seated and after a minimum of 5 minutes of rest. The mean blood pressure of the right arm was measured thrice for each participant, with a 1-minute interval between each measurement, and was utilized in the final analysis. The fasting venous blood samples of all participants were collected by uniformly trained nurses and subsequently transferred to Biaojia Biotechnology in Shenzhen, China for laboratory analyses utilizing automatic clinical analyzers (Beckman Coulter, USA). The biochemical data encompassed measurements of plasma total cholesterol (TC), TG, HDL-C, LDL-C, and homocysteine (Hcy), as well as serum aspartate aminotransferase (AST), alanine aminotransferase (ALT), uric acid (UA), albumin, total bilirubin (TBiL), direct bilirubin (DBiL), and creatinine. The estimated glomerular filtration rate (eGFR) was determined using the Chronic Kidney Disease Epidemiological Collaboration equation (27).

### 2.3 Definitions

T2DM was characterized by a fasting glucose level of ≥ 7 mmol/L, a self-reported history of diabetes, or the use of hypoglycemic therapy (28). The definition of the Atherogenic Index of Plasma (AIP) was derived from the base-10 logarithm of the TG/HDL-C ratio (29). Hyperuricemia was diagnosed as serum UA levels > 420 μmol/L (7 mg/dL), regardless of sex (30).

### 2.4 Statistical analysis

The baseline characteristics of the study participants were presented as mean ± standard deviation (SD) for continuous variables and number (%) for categorical variables. The data were analyzed via ANOVA for continuous variables and Chi-square test for categorical variables to identify discerning any disparities between groups based on AIP quartiles. The distribution of AIP values ​​of the subjects was normal. Multivariate logistic regression analyses were performed to assess the association between AIP and T2DM (odds ratio [OR] and 95% confidence interval [CI]). P values for the trends of groups by AIP quartiles were calculated to confirm the existence of a linear correlation between AIP and T2DM. Fourmodels were constructed to examine the correlation between AIP and T2DM under varying states of uric acid levels: crude model, unadjusted; model 1, adjusted for age, sex, WC, SBP, DBP; model 2, adjusted for age, sex, WC, SBP, DBP, current smoking, current drinking, education level, physical activity, Hcy, TC, LDL-C, AST, ALT, TBiL, DBiL, albumin, eGFR, stroke, and antihypertensive drugs; Model 3 was adjusted for age, sex, WC, BMI, SBP, DBP, current smoking, current drinking, education level, physical activity, Hcy, TC, LDL-C, AST, ALT, TBiL, DBiL, albumin, eGFR, stroke, and antihypertensive drugs.

The selection of covariates for adjustment was predicated on their clinical significance, univariable analysis, and their capacity to modify the OR values of T2DM by a minimum threshold of 10%. Additionally, a Generalized Additive Model (GAM) coupled with a penalized spline method was employed to construct a smooth fitting curve, thereby facilitating a visual evaluation of the dose-response correlation between AIP and T2DM. Upon detection of nonlinearity, a recursive algorithm was initially employed to compute the inflection point. Subsequently, a two-stage logistic regression model was constructed on either side of the inflection point. The log-likelihood ratio served as a determinant for identifying the most appropriate model to elucidate the association between AIP and T2DM. A sequence of sensitivity analyses was conducted to incrementally ascertain the reliability of the conclusions. Given the potential influence of gender and age on the prevalence of T2DM, additional multiple logistic regression analyses were executed to discern the correlation between AIP and T2DM, stratified by gender and age. In addition, E-values were calculated to assess for the effect of unmeasured confounders on the association between AIP and T2DM. Subsequently, stratified analyses were conducted to explore the potential variables that could modified the association between AIP and T2DM.

The statistical analyses were carried out utilizing R version 4.2.0 (http://www.r-project.org) and EmpowerStats (www.empowerstats.net; X&Y Solutions, Inc. Boston, Massachusetts). A statistically significant difference was determined by a two-sided *P*-value < 0.05.

## 3 Results

### 3.1 Baseline characteristic

The present study comprised a cohort of 8,258 normal-weight adults with hypertension. The average age was 64.89 ± 8.97 years and 48.60% of participants were male. The average baseline value of the AIP index was -0.04 ± 0.29. The overall prevalence of T2DM in the cohort population was 15.55%. **Table 1** presents the baseline characteristics of the participants, categorized according to quartiles of AIP. There was a progressive escalation in the prevalence of T2DM as the AIP increased, with respective percentages of 10.78% (221), 12.76% (251), 14.78% (305), and 24.55% (507). In comparison to the lower AIP group, the higher AIP group demonstrated increased levels of WC, BMI, TC, TG, LDL-C, ALT, UA, and Albumin. Furthermore, the higher AIP group displayed a higher prevalence of stroke and a superior level of educational achievement. Conversely, the group with lower AIP exhibited higher values for age, Hcy, TBiL, DBiL, HDL-C, and eGFR when compared to the higher AIP group. Concurrently, the lower AIP group had a higher proportion of males, smoking, alcohol consumption, and high physical activity. Notably, there was no significant difference in systolic blood pressure and antihypertensive medication between the four groups. Eventually, the baseline characteristics of the participants were presented in **Supplementary Table S1**, stratified by uric acid (UA) concentration.

**Table 1:** Baseline characteristics of the study population according to AIP index.

	Q1	Q2	Q3	Q4	*P*-value
N	2,065	2,064	2,064	2,065	
Male, n (%)	1,250 (60.53)	1,015 (49.18)	885 (42.88)	863 (41.79)	< 0.001
Age, years	66.06 ± 8.74	66.00 ± 9.04	64.71 ± 8.76	62.77 ± 8.94	< 0.001
WC, cm	77.59 ± 6.56	79.62 ± 6.49	81.56 ± 7.10	83.04 ± 6.11	< 0.001
BMI, kg/m^2^	21.49 ± 1.72	21.89 ± 1.74	22.36 ± 1.69	22.75 ± 1.57	< 0.001
Education, n (%)					< 0.001
< High school	1973 (95.54)	1938 (93.90)	1933 (93.65)	1877 (90.90)	
≥ High school	92 (4.46)	126 (6.10)	131 (6.35)	188 (9.10)	
Current smoking, n (%)	681 (32.98)	575 (27.86)	503 (24.37)	526 (25.48)	< 0.001
Current drinking, n (%)	681 (32.98)	444 (21.52)	380 (18.41)	355 (17.20)	< 0.001
Physical activity, n (%)					0.002
Low	1067 (51.67)	1152 (55.80)	1123 (54.39)	1191 (57.67)	
Moderate	486 (23.54)	490 (23.73)	499 (24.18)	486 (23.55)	
high	512 (24.79)	422 (20.47)	442 (21.42)	388 (18.78)	
SBP, mmHg	148.84 ± 17.75	148.95 ± 18.21	149.03 ± 17.49	148.08 ± 18.02	0.249
DBP, mmHg	88.13 ± 10.70	88.01 ± 10.59	88.19 ± 10.11	89.26 ± 10.43	< 0.001
Serum homocysteine, μmol/L	17.93 ± 10.12	18.77 ± 11.57	18.45 ± 11.85	17.54 ± 10.25	< 0.001
Serum uric acid, μmol/L	398.32 ± 114.54	399.08 ± 114.62	413.82 ± 117.31	435.62 ± 123.53	< 0.001
Serum AST, U/L	26.88 ± 10.34	25.72 ± 10.94	25.71 ± 11.46	25.94 ± 13.12	< 0.001
Serum ALT, U/L	16.87 ± 9.78	17.58 ± 11.99	18.66 ± 12.07	20.57 ± 15.56	< 0.001
TBiL, μmol/L	15.52 ± 9.10	14.39 ± 6.24	14.07 ± 6.34	13.90 ± 6.20	< 0.001
DBiL, μmol/L	6.29 ± 4.26	5.55 ± 2.04	5.25 ± 2.11	5.00 ± 2.15	< 0.001
Albumin, g/L	46.29 ± 4.28	46.28 ± 4.04	46.58 ± 4.00	47.01 ± 3.94	< 0.001
Total cholesterol, mmol/L	5.10 ± 1.01	5.13 ± 1.07	5.24 ± 1.11	5.18 ± 1.16	< 0.001
Triglyceride, mmol/L	0.83 ± 0.12	1.20 ± 0.25	1.63 ± 0.36	2.97 ± 1.51	< 0.001
LDL-C, mmol/L	2.70 ± 0.72	2.91 ± 0.77	3.10 ± 0.80	3.11 ± 0.80	< 0.001
HDL-C, mmol/L	2.00 ± 0.43	1.68 ± 0.34	1.49 ± 0.29	1.26 ± 0.28	< 0.001
eGFR (ml/min per 1.73 m^2^)	89.03 ± 18.88	86.66 ± 19.79	86.68 ± 20.14	87.72 ± 20.55	< 0.001
Self-reported stroke, n (%)	102 (4.94)	137 (6.64)	147 (7.12)	144 (6.97)	0.015
Self-reported diabetes, n (%)	221 (10.78)	251 (12.76)	305 (14.78)	507 (24.55)	< 0.001
Antihypertensive drugs, n (%)	1,270 (61.50)	1,311 (63.52)	1,309 (63.42)	1,338 (64.83)	0.172

Data are presented as the mean ± SD, or number (percentage).

WC, waist circumference; BMI, body mass index; SBP, systolic blood pressure; DBP, diastolic blood pressure; AST, aspartate aminotransferase; ALT, alanine aminotransferase; TBiL, total bilirubin; DBiL, direct bilirubin; eGFR, estimated glomerular filtration rate; LDL-C, Low-density lipoprotein cholesterol; HDL-C, High-density lipoprotein cholesterol; AIP, atherogenic index of plasm.

### 3.2 The relationship between AIP and T2DM

Overall, there was a positive correlation between AIP and T2DM irrespective of the adjustment for confounders. The findings of the multivariate logistical regression analysis elucidating the relationship between AIP and T2DM are presented in **Table 2**. Model 3 represents the fully adjusted model, which accounted for a plenty of confounders such as age, sex, WC, BMI, SBP, DBP, current smoking and drinking habits, education level, physical activity, Hcy, TC, LDL-C, AST, ALT, TBiL, DBiL, Albumin, eGFR, stroke, and usage of antihypertensive drugs. In the fully adjusted model, the odds ratio (OR) for T2DM was determined to be 3.73 (95% CI: 2.82, 4.94). When AIP was transformed into categorical variables based on quartile, the adjusted ORs for T2DM in the quartiles 2, quartiles 3, and quartiles 4 were 0.93 (95% CI: 0.74, 1.18), 1.08 (95% CI: 0.85, 1.37), and 1.99 (95% CI: 1.57, 2.52), respectively, in comparison to the quartiles 1 in Model 3 (*P* for trend < 0.001). **Table 3** elucidates the correlation between AIP and T2DM, stratified by hyperuricemia. In the fully adjusted model, per unit increment of AIP corresponds to a 5.36-fold increase in T2DM risk among the hyperuricemia population. Similarly, AIP was divided into quartiles for further analysis. Using the quartile 1 as the reference group, the quartiles 2 and 3 did not exhibit a significant increase in T2DM risk, with ORs of 1.02 (95% CI: 0.72, 1.44) and 1.31 (95% CI: 0.93, 1.85), respectively. However, there was a significantly higher prevalence of T2DM in the quartile 4 (OR: 2.79; 95% CI: 1.98, 3.94). Conversely, within the population exhibiting normal uric acid levels, the impact of AIP on T2DM is comparatively mild (OR: 2.63; 95% CI: 1.77, 3.90). A 42% heightened risk of T2DM was observed in quartiles 4 relative to the quartiles 1. Noteworthily, there was no significant correlation between AIP and T2DM in the quartiles 2 and quartiles 3, exhibiting ORs of 0.87 (95% CI: 0.63, 1.20) and 0.88 (95% CI: 0.64, 1.21) respectively. The aforementioned findings imply a potential interaction between hyperuricemia and AIP (*P* for interaction = 0.034).

**Table 2:** Association between AIP and T2DM in different models.

AIP Index		T2DM, OR (95%CI)			
	Events, n (%)	Crude Model	Model 1	Model 2	Model 3
Continuous					
Per 1 unit increment	1,284 (15.55)	4.24 (3.47, 5.18)	3.37 (2.72, 4.19)	3.76 (2.84, 4.97)	3.73 (2.82, 4.94)
Categories, quartiles					
Q1 (< -0.24)	221 (10.70)	Reference		Reference	Reference
Q2 (-0.24 to < -0.06)	251 (12.16)	1.16 (0.95, 1.40)	1.03 (0.85, 1.25)	0.93 (0.74, 1.18)	0.93 (0.74, 1.18)
Q3 (-0.06 to < 0.14)	305 (14.78)	1.45 (1.20, 1.74)	1.18 (0.97, 1.42)	1.08 (0.85, 1.37)	1.08 (0.85, 1.37)
Q4 (≥ 0.14)	507 (24.55)	2.72 (2.29, 3.22)	2.14 (1.79, 2.57)	2.00 (1.58, 2.53)	1.99 (1.57, 2.52)
*P* for trend		< 0.001	< 0.001	< 0.001	< 0.001

Crude Model was adjusted for none.

Model 1 was adjusted for age, sex, WC, SBP, DBP.

Model 2 was adjusted for age, sex, WC, SBP, DBP, current smoking, current drinking, education level, physical activity, Hcy, TC, LDL-C, AST, ALT, TBiL, DBiL, albumin, eGFR, stoke, and antihypertensive drugs.

Model 3 was adjusted for age, sex, WC, BMI, SBP, DBP, current smoking, current drinking, education level, physical activity, Hcy, TC, LDL-C, AST, ALT, TBiL, DBiL, albumin, eGFR, stroke, and antihypertensive drugs.

**Table 3:** Association between AIP and T2DM in different models stratified by hyperuricemia.

AIP Index		T2DM, OR (95%CI)			
	Events, n (%)	Crude Model	Model 1	Model 2	Model 3
hyperuricemia					
Per 1 unit increment	597 (17.17)	5.03 (3.76, 6.75)	3.92 (2.84, 5.40)	5.45 (3.67, 8.10)	5.36 (3.61, 7.98)
Quartiles					
Q1 (< -0.21)	101 (11.62)	Reference		Reference	Reference
Q2 (-0.21 to < -0.01)	101 (11.62)	1.00 (0.75, 1.34)	0.89 (0.66, 1.21)	1.02 (0.72, 1.44)	1.02 (0.72, 1.44)
Q3 (-0.01 to < 0.19)	146 (16.80)	1.54 (1.17, 2.02)	1.26 (0.94, 1.67)	1.32 (0.94, 1.86)	1.31 (0.93, 1.85)
Q4 (≥ 0.19)	249 (28.65)	3.05 (2.37, 3.94)	2.38 (1.81, 3.14)	2.83 (2.00, 3.99)	2.79 (1.98, 3.94)
*P* for trend		< 0.001	< 0.001	< 0.001	< 0.001
non-hyperuricemia					
Per 1 unit increment	687 (14.37)	3.45 (2.61, 4.57)	2.58 (1.90, 3.50)	2.62 (1.77, 3.89)	2.63 (1.77, 3.90)
Quartiles					
Q1 (< -0.26)	126 (10.54)	Reference	Reference	Reference	Reference
Q2 (-0.26 to < -0.09)	150 (12.55)	1.22 (0.95, 1.57)	1.08 (0.83, 1.39)	0.87 (0.63, 1.20)	0.87 (0.63, 1.20)
Q3 (-0.09 to < 0.10)	163 (13.64)	1.34 (1.05, 1.72)	1.06 (0.82, 1.37)	0.88 (0.64, 1.21)	0.88 (0.64, 1.21)
Q4 (≥ 0.10)	248 (20.74)	2.22 (1.76, 2.80)	1.67 (1.31, 2.14)	1.42 (1.03, 1.96)	1.42 (1.03, 1.96)
*P* for trend		< 0.001	< 0.001	0.007	0.007
*P* value for interaction		0.055	0.006	0.033	0.034

Crude Model was adjusted for none.

Model 1 was adjusted for age, sex, WC, SBP, DBP.

Model 2 was adjusted for age, sex, WC, SBP, DBP, current smoking, current drinking, education level, physical activity, Hcy, TC, LDL-C, AST, ALT, TBiL, DBiL, albumin, eGFR, stoke, and antihypertensive drugs.

Model 3 was adjusted for age, sex, WC, BMI, SBP, DBP, current smoking, current drinking, education level, physical activity, Hcy, TC, LDL-C, AST, ALT, TBiL, DBiL, albumin, eGFR, stroke, and antihypertensive drugs.

### 3.3 Sensitivity analyses

To verify the robustness of the findings, several sensitivity analyses were conducted. Despite the fact that this study adjusted for a substantial number of confounding variables, there remains the potential for unmeasured confounders that could affect the association between AIP and T2DM. Consequently, an E-value was computed to evaluate the potential influence of these unmeasured confounders on the study’s results. The E-value (3.29) derived from this study notably exceeded the relative risk (2.33) correlated with AIP and unmeasured confounders. This implies that the influence of unmeasured confounders on the relationship between AIP and T2DM is not sufficient to generate a false-positive outcome.

Considering that age and gender could potentially influence the relationship between AIP and T2DM, we conducted sensitivity analyses, which were stratified by age (**Supplementary Table S2**). Multiple logistic regression analyses indicated a positive association between AIP and the risk of T2DM in both the group aged < 65 years (OR: 3.37; 95% CI: 2.26, 5.01) and the group aged ≥ 65 years (OR: 4.54; 95% CI: 3.05, 6.75). Similarly, multivariate logistic regression analyses were implemented to examine the correlation between AIP and T2DM, stratified by gender (**Supplementary Table S3**). The findings revealed that the association between AIP and T2DM persisted consistently in both male (OR: 4.74; 95% CI: 3.00, 7.50) and female (OR: 3.20; 95% CI: 2.26, 4.55).

### 3.4 The J-shaped relationship between AIP and T2DM

**Figure 1:**
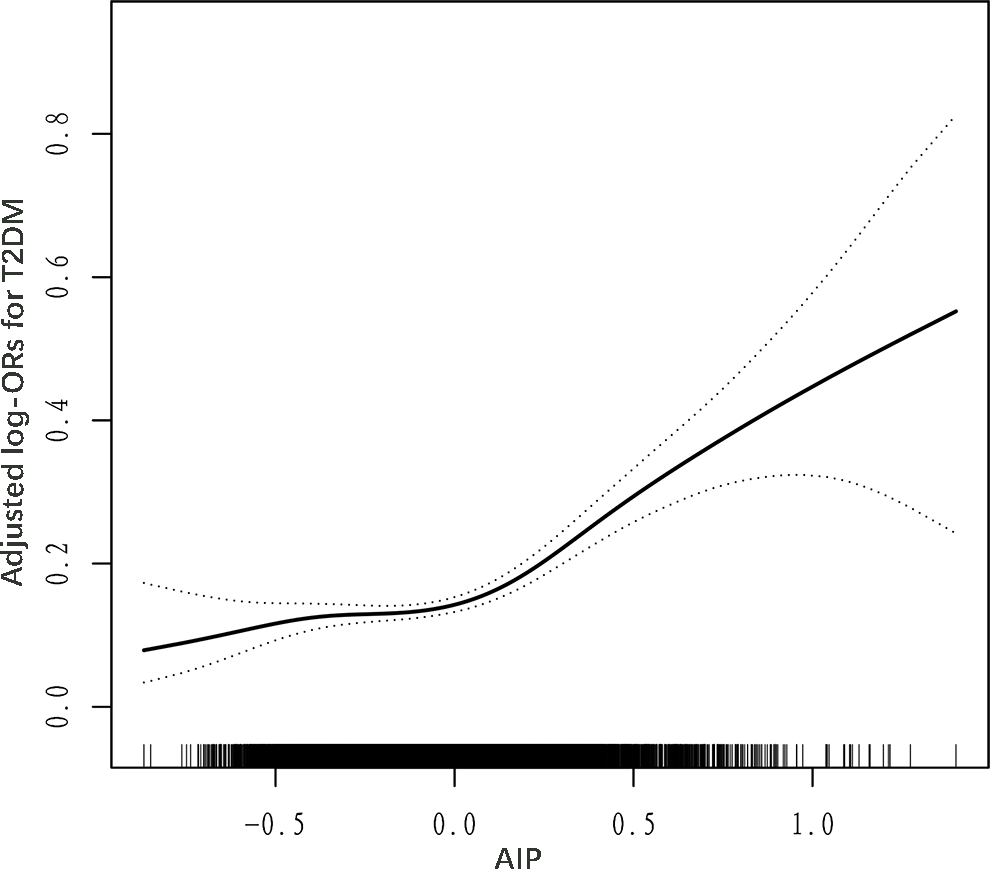
Dose–response relationship between AIP and T2DM. Adjusted for age, sex, WC, SBP, DBP, education, physical activity, current smoking, current drinking, Hcy, AST, ALT, TBiL, DBiL, UA, albumin, TC, LDL-C, eGFR, stroke, and antihypertensive drugs.

Generalized additive models (GAM) and fitted smoothing curves (penalized spline method) were performed to visually assess the correlation between AIP and the risk of T2DM. After adjusting for relevant confounders, a J-shaped association was detected between AIP and T2DM, with a inflection point of 0 identified by recursive algorithm (**Figure 1**; **Table 4**). when AIP was > 0, AIP was independently and positively associated with the risk of T2DM (OR: 6.20; 95% CI: 4.20, 9.14). conversely, when AIP was < 0, there was no significant correlation between AIP and T2DM (OR: 1.37; 95% CI: 0.75, 2.51).

**Table 4:** Threshold effect analyses of AIP on T2DM by a two-piecewise logistic regression model.

AIP	Adjusted OR (95% CI), P value
model I	
Fitting by the standard linear model	3.76 (2.84, 4.97), < 0.001
model II	
Inflection point	0
< 0	1.37 (0.75, 2.51), 0.308
> 0	6.20 (4.20, 9.14), < 0.001
Log likelihood ratio	< 0.001

Adjusted for age, sex, WC, SBP, DBP, current smoking, current drinking, education level, physical activity, Hcy, TC, LDL-C, AST, ALT, TBiL, DBiL, albumin, eGFR, stroke, and antihypertensive drugs.

### 3.5 Subgroup analysis

**Figure 2:**
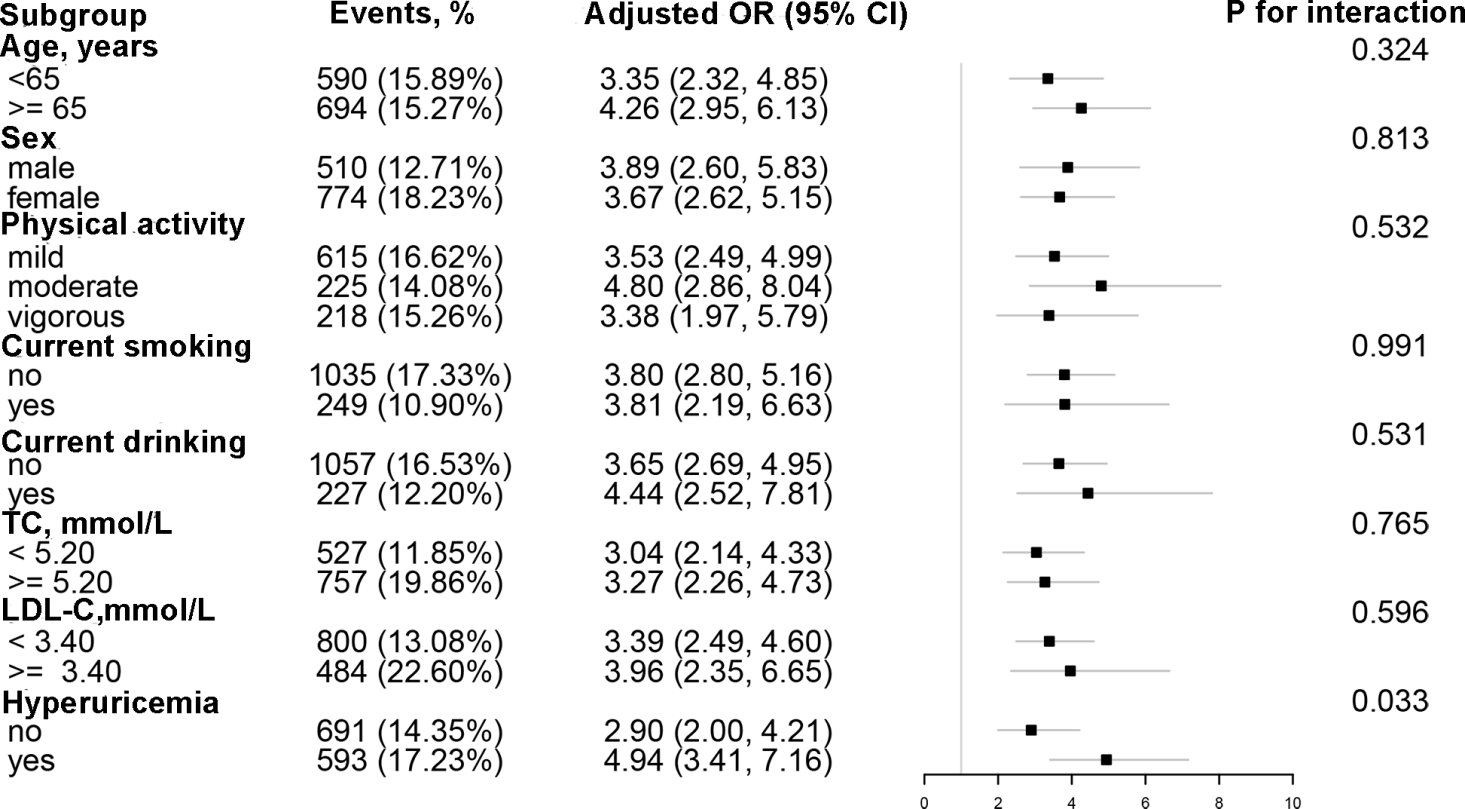
Subgroup analyses of the effect of AIP on T2DM. Each subgroup analysis adjusted for age, sex, WC, SBP, DBP, education, physical activity, current smoking, current drinking, Hcy, AST, ALT, TBiL, DBiL, UA, albumin, TC, LDL-C, eGFR, stroke, and antihypertensive drugs, except for the stratifying variable.

Stratified analyses were conducted to detect potential variables that could modified the relationship between AIP and the risk of T2DM (**Figure 2**) The correlation between AIP and T2DM remained consistent across the following subgroups: age (< 65 vs. ≥ 65 year), sex (male vs. ≥ female), physical activity (mild, moderate, vigorous), current smoking status (no vs. yes), drinking status (no vs. yes), TC (< 5.20 vs. ≥ 5.20 mmol/L), and LDL-C (< 2.6 vs. ≥ 2.6 mmol/L). Nevertheless, the association between AIP and T2DM was significantly stronger in the hyperuricemia population compared to individuals with normal uric acid levels (*P* for interaction = 0.034).

## 4 Discussion

The current study analyzed the data from the China Hypertension Registry Study and found that AIP was positively correlated with the risk of T2DM among normal-weight adults with hypertension. Notably, a J-shaped association was detected between AIP and T2DM, with an inflection point of 0. Furthermore, the relationship between AIP and T2DM was more pronounced in hyperuricemia population compared to individuals with normal uric acid.

A cross-sectional study encompassing 9,764 Chinese participants demonstrated an independent positive correlation between AIP and the risk of T2D (31). A longitudinal study conducted by Tohidi et al., which tracked 5,064 Iranian subjects over a span of more than a decade, revealed a significant escalation in the incidence of T2DM among the population with elevated TG/HDL-C levels. This correlation remained consistent even after adjusting for pertinent confounding factors (32). Previous studies predominantly observed the relationship between baseline AIP and the incidence of T2DM. However, the influence of longitudinal changes in AIP on the development of T2DM remains unclear. Yi et al. conducted a long-term follow-up study involving 8,760 middle-aged and elderly Chinese participants. Their findings indicated that, in comparison to maintaining a low AIP pattern, the hazard ratios (HRs) for maintaining a high AIP, transitioning from high to low AIP, and transitioning from low to high AIP were 1.69, 1.32, and 1.47, respectively (all *P* < 0.05). Furthermore, when compared to the group maintaining a high AIP, the risk of T2DM in the high to low AIP group did not show a significant reduction (33). In alignment with the aforementioned research findings, the present study also identified a positive correlation between AIP and T2DM, irrespective of adjustments made for relevant confounding variables. However, it is noteworthy that the majority of the preceding studies were primarily focused on individuals exhibiting a relatively low risk of cardiovascular disease. In contrast, this study incorporated patients diagnosed with hypertension, a demographic characterized by a high risk of cardiovascular disease. This inclusion broadens the applicability of AIP in assessing the risk of T2DM and may offer novel perspectives on lipid management objectives within the context of hypertension. It is widely recognized that obesity is a significant risk factor for T2DM. Nevertheless, there exists a subset of normal-weight individuals who exhibit metabolic disorders similar to those observed in obese individuals. These individuals, termed as ‘metabolically obese’, constitute approximately 20% of the normal-weight population (34). Prior studies were predominantly conducted on the general population and individuals with obesity, resulting in an ambiguous comprehension of the relationship between AIP and T2DM in normal-weight individuals. This study addressed this knowledge gap, and demonstrated that elevated AIP increased the risk of T2DM independently of weight. These findings further support the theory of metabolic obesity.

Gender interaction is a prevalent concept in clinical research. Previous studies have documented the impact of AIP gender-interaction on T2DM. Shi et al. extracted data from the 2011-2018 National Health and Nutrition Examination Survey (NHANES) database. The findings revealed a significant positive correlation between AIP and the prevalence of T2DM in females, but this relationship was not observed in males (*P* for interaction < 0.0001) (35). Similarly, a prospective cohort study involving 5,201 non-diabetic Iranian participants indicated that the association between TG/HDL-C ratio and the incidence of T2DM was more pronounced in women than in men (36). In contrast, the present study found that the effect size of the AIP on T2DM was greater in males compared to females (*P* for interaction = 0.813). The discrepancies observed in the aforementioned conclusions can be ascribed to variations in study populations, sample sizes, and controlled confounding variables. Given that the average age of participants in this study exceeded 60 years, the majority of the women were postmenopausal. During menopause, there is a marked decline in estrogen levels accompanied by relatively elevated androgen levels. These hormonal alterations can result in an increase in pro-inflammatory factors and free fatty acids, which may subsequently lead to insulin resistance and hyperinsulinemia (37). The markedly higher prevalence of T2DM observed in women compared to men in this study further corroborates this perspective. However, the differences in baseline characteristics between male and female participants must be acknowledged. Specifically, male participants exhibited greater age, waist circumference, Hcy levels, and uric acid levels, in addition to higher rates of smoking and alcohol consumption (**Supplementary Table S4**), all of which are established as the risk factors of T2DM (38–41). Generally, the male cohort in this study exhibited a higher prevalence of metabolic-related risk factors compared to their female counterparts, potentially elucidating the more pronounced association between AIP and T2DM risk observed in men. Although no interaction between age, gender, and AIP was identified in relation to T2DM, this study is the first to reveal the modifying effect of hyperuricemia on the AIP-T2DM relationship. Specifically, the impact of AIP on T2DM risk was found to be more substantial in individuals with hyperuricemia than in those with normal uric acid levels. Uric acid is a byproduct of the purine degradation pathway. Numerous epidemiological studies across diverse populations have identified hyperuricemia as an independent risk factor for T2DM (42–44). The mechanisms through which hyperuricemia contributes to the development of T2DM include: inhibition of nitric oxide production and utilization by endothelial cells, resulting in hyperinsulinemia (45); enhancement of intracellular reactive oxygen species production, exacerbating insulin resistance in adipocytes (46); and promotion of inflammatory processes, leading to β-cell dysfunction (47). It is noteworthy that a potential interaction exists between uric acid and T2DM. Hyperinsulinemia may contribute to elevated uric acid levels by promoting increased reabsorption and decreased excretion of uric acid in the proximal renal tubules (48). Consequently, the findings of this study could enhance the precision of AIP management in clinical practice. Specifically, for normal-weight patients presenting with hypertension and hyperuricemia, more rigorous AIP management protocols may be required to mitigate the risk of T2DM.

The present study identified a J-shaped relationship between AIP and T2DM in normal-weight Chinese adults with hypertension, with an inflection point at 0. This J-shaped correlation has been corroborated by prior research. Specifically, a cross-sectional study involving 12,060 Chinese participants aged 45 years or older demonstrated a J-shaped association between AIP and the prevalence of T2DM, with an inflection point at -0.04 (49). Similarly, Yin et al. performed a cross-sectional analysis recruiting 9,245 American adults, identifying a threshold effect between AIP and the risk of T2DM. Their findings indicated that when AIP exceeded -0.47, the risk of T2DM increased significantly with rising AIP levels. Conversely, when AIP was below -0.47, no significant correlation was observed between AIP and T2DM (50). Compared to the aforementioned studies, the inflection point value observed in the present research is relatively high. This discrepancy may be attributed to the heterogeneity of the participants across different studies. Specifically, the subjects in this study were individuals of normal weight, whereas the other studies encompassed general populations. Additionally, racial differences may also account for this variation. For instance, the study by Yin et al. involved American adults, whereas the current research focused on Chinese adults. Prediabetes denotes a condition characterized by elevated blood glucose levels that do not meet the diagnostic criteria for diabetes, characterized by insulin resistance (51). Approximately 70% of individuals with prediabetes will eventually progress to T2DM (52). We hypothesize that an increase in the Atherogenic Index of Plasma (AIP) is positively associated with an elevated risk of prediabetes, while the risk of progressing to T2DM remains relatively unaffected until a certain AIP threshold is reached. Beyond this threshold, AIP is significantly positively correlated with the risk of T2DM. This hypothesis has been preliminarily supported by previous studies (50, 53). The identification of the inflection point possesses substantial clinical significance, as it provides clinicians with precise intervention target values ​​for AIP. Early intervention based on these AIP values has the potential to significantly mitigate the risk of T2DM. It is crucial to emphasize that the inflection point of AIP may vary among individuals with different body weights. Consequently, further research is warranted to ascertain the specific inflection points for populations with diverse weight profiles.

### 4.1 Limitations and strengths

It is essential to recognize both the strengths and limitations of the present study when interpreting our findings. To our knowledge, the current study is the first to investigate the relationship between AIP and the risk of T2DM in normal-weight adults with hypertension. Furthermore, in assessing the independent association between AIP and T2DM, this study accounted for a substantial number of confounding variables and evaluated the potential influence of unknown confounders by calculating the E value. This methodological approach was employed to mitigate the impact of confounding factors on the results to the greatest extent possible. Additionally, stratified analyses were conducted to assess the robustness of the findings and to identify potential effect modifiers that may influence the correlation between AIP and T2DM. Nonetheless, it is imperative to acknowledge several limitations inherent in this study. Primarily, the cross-sectional design was insufficient to establish a causality between AIP and T2DM. Secondly, the diagnostic criterion utilized in this study was fasting plasma glucose. However, measurements of glycosylated hemoglobin (HbA1c) levels were not undertaken, nor were oral glucose tolerance tests (OGTT) administered. Consequently, there is a potential for diagnostic misclassification, whereby some diabetic patients may not have been accurately identified. This limitation could lead to an underestimation of the association between AIP and T2DM. It is noteworthy that the Framingham Heart Study also employed fasting blood glucose levels as the method for diagnosing T2DM (54). Thirdly, although the current study adjusted for a substantial number of confounding variables to mitigate their impact on the research outcomes, the inherent limitations of cross-sectional studies cannot entirely preclude the influence of unmeasured confounding factors. Nevertheless, through the calculation of the E-value, it was determined that the impact of these unknown confounders on the study’s results is negligible. Finally, the study’s sample comprised normal-weight Chinese adults with hypertension, which restricts the generalizability of the findings to other populations.

## 5 Conclusion

This cross-sectional study involving 8,258 normal-weight adults with hypertension identified a J-shaped relationship between AIP and T2DM, with an inflection point at 0. Furthermore, an interaction between hyperuricemia and AIP was observed, indicating that the association between AIP and T2DM was more pronounced in individuals with hyperuricemia compared to those with normal uric acid. Consequently, AIP may serve as a simple and effective index for assessing the risk of T2DM in routine clinical practice. It is worth noting that the normal reference value of AIP may vary across different populations. Therefore, further research within diverse demographic is necessary.

## Data Availability

The raw data supporting the conclusions of this article will be made available by the authors, without undue reservation.
