# Supplementary material for: Threshold effect of atherogenic index of plasma on type 2 diabetes mellitus and modification by uric acid in normal-weight adults with hypertension
Source: Front Endocrinol (Lausanne). 2024 Nov 27;15:1495340. doi: 10.3389/fendo.2024.1495340 (PMC11631599; doi:10.3389/fendo.2024.1495340)
Supplement: Supplementary file 1 [file DataSheet1.docx]

**Table S1 Baseline characteristics of the study population according to hyperuricemia**

| Variables | Total = 8,258 | Non-hyperuricemia = 4,817 | Hyperuricemia = 3,441 | P-value |
| --- | --- | --- | --- | --- |
| Male, n (%) | 4,013（48.60） | 1,684 (34.96) | 2,329 (67.68) | < 0.001 |
| Age, years | 64.89 ± 8.97 | 64.31 ± 8.73 | 65.70 ± 9.22 | < 0.001 |
| WC, cm | 80.45 ± 6.88 | 79.66 ± 6.78 | 81.56 ± 6.87 | < 0.001 |
| Education, n (%) |  |  |  | < 0.001 |
| < High school | 7,701 (93.26) | 4,618 (95.87) | 3,103 (90.18) |  |
| ≥ High school | 557 (6.74) | 199 (4.13) | 338 (9.82) |  |
| Current smoking, n (%) | 2,285 (27.67) | 1,062 (22.05) | 1,223 (35.55) | < 0.001 |
| Current drinking, n (%) | 1,860 (22.52) | 789 (16.38) | 1,071 (31.13) | < 0.001 |
| Physical activity, n (%) |  |  |  | 0.008 |
| Low | 4,540 (54.98) | 2,569 (53.33) | 1,964 (57.08) |  |
| Moderate | 1,962 (23.76) | 1,194 (24.79) | 773 (22.48) |  |
| high | 1,756 (21.26) | 1,054 (21.89) | 704 (20.45) |  |
| SBP, mmHg | 148.72 ± 17.87 | 149.49 ± 17.29 | 147.65 ± 18.60 | < 0.001 |
| DBP, mmHg | 88.40 ± 10.47 | 88.37 ± 10.14 | 88.43 ± 10.92 | 0.782 |
| Serum homocysteine, μmol/L | 18.17 ± 10.98 | 16.50 ± 9.11 | 20.51 ± 12.81 | < 0.001 |
| Serum AST, U/L | 26.06 ± 11.52 | 24.93 ± 10.49 | 27.64 ± 12.66 | < 0.001 |
| Serum ALT, U/L | 18.42 ± 12.60 | 17.32 ± 11.78 | 19.96 ± 13.51 | < 0.001 |
| TBiL, μmol/L | 14.47 ± 7.10 | 14.18 ± 7.28 | 14.88 ± 6.83 | < 0.001 |
| DBiL, μmol/L | 5.52 ± 2.84 | 5.44 ± 3.18 | 5.63 ± 2.27 | < 0.001 |
| Albumin, g/L | 46.54 ± 4.08 | 46.64 ± 4.07 | 46.41 ± 4.09 | < 0.001 |
| Total cholesterol, mmol/L | 5.16 ± 1.09 | 5.14 ± 1.06 | 5.19 ± 1.14 | 0.148 |
| Triglyceride, mmol/L | 1.66 ± 1.13 | 1.56 ± 0.99 | 1.79 ± 1.29 | < 0.001 |
| LDL-C, mmol/L | 2.95 ± 0.79 | 2.93 ± 0.77 | 2.98 ± 0.82 | 0.02 |
| HDL-C, mmol/L | 1.61 ± 0.44 | 1.63 ± 0.43 | 1.57 ± 0.44 | < 0.001 |
| AIP | -0.04 ± 0.29 | -0.06 ± 0.28 | -0.00 ± 0.30 | < 0.001 |
| eGFR (ml/min per 1.73 m^2^) | 87.52 ± 19.87 | 93.93 ± 15.40 | 78.56 ± 21.86 | < 0.001 |
| Self-reported stroke, n (%) | 530 (6.42) | 293 (6.08) | 237 (6.89) | 0.141 |
| Self-reported diabetes, n (%) | 1284 (15.55) | 691 (14.35) | 593 (17.23) | < 0.001 |
| Antihypertensive drugs, n (%) | 5,228 (63.31) | 2,953 (61.30) | 2,275 (66.13) | < 0.001 |

Data are presented as the mean ± SD, or number (percentage)

Abbreviations: WC waist circumference, SBP systolic blood pressure, DBP diastolic blood pressure, AST aspartate aminotransferase, ALT alanine aminotransferase, TBiL total bilirubin, DBiL direct bilirubin, eGFR estimated glomerular filtration rate, LDL-C Low-density lipoprotein cholesterol, HDL-C High-density lipoprotein cholesterol, AIP atherogenic index of plasm.

**Table S2 Association between AIP and T2DM in different models stratified by age**

| AIP Index |  | T2DM, OR (95%CI) | |  |  |
| --- | --- | --- | --- | --- | --- |
|  | Events, n (%) | Crude Model | Model 1 | Model 2 | Model 3 |
| Age < 65 years |  |  |  |  |  |
| Per 1 unit increment | 590 (15.89) | 4.21 (3.15, 5.62) | 3.76 (2.75, 5.15) | 3.37 (2.27, 5.02) | 3.37 (2.26, 5.01) |
| Quartiles |  |  |  |  |  |
| Q1 (< -0.20) | 99 (10.66) | Reference |  | Reference | Reference |
| Q2 (-0.20 to < -0.02) | 112 (12.07) | 1.15 (0.96, 1.53) | 1.00 (0.75, 1.35) | 0.82 (0.57, 1.17) | 0.82 (0.57, 1.17) |
| Q3（-0.02 to < 0.19） | 146 (15.73) | 1.57 (1.19, 2.06) | 1.35 (1.01, 1.79) | 1.09 (0.77, 1.55) | 1.09 (0.77, 1.55) |
| Q4（≥ 0.19） | 233 (25.08) | 2.81 (2.17, 3.62) | 2.38 (1.81, 3.13) | 1.90 (1.34, 2.68) | 1.90 (1.34, 2.68) |
| P for trend |  | < 0.001 | < 0.001 | < 0.001 | < 0.001 |
| Age ≥ 65 years |  |  |  |  |  |
| Per 1 unit increment | 694 (15.27) | 4.38 (3.30, 5.82) | 3.09 (2.29, 4.18) | 4.57 (3.07, 6.79) | 4.54 (3.05, 6.75) |
| Quartiles |  |  |  |  |  |
| Q1 (< -0.26) | 124 (10.92) | Reference |  | Reference | Reference |
| Q2 (-0.26 to < -0.09) | 146 (12.85) | 1.20 (0.93, 1.55) | 1.07 (0.82, 1.38) | 1.07 (0.78, 1.47) | 1.07 (0.78, 1.47) |
| Q3（-0.09 to < 0.10） | 158 (13.91) | 1.32 (1.03, 1.69) | 1.04 (0.80, 1.35) | 1.07 (0.78, 1.48) | 1.07 (0.78, 1.48) |
| Q4（≥ 0.10） | 266 (23.42) | 2.50 (1.98, 3.15) | 1.83 (1.43, 2.33) | 2.06 (1.49, 2.86) | 2.05 (1.48, 2.85) |
| P for trend |  | < 0.001 | < 0.001 | < 0.001 | < 0.001 |
| P value for interaction |  | 0.843 | 0.718 | 0.324 | 0.338 |

Crude Model was adjusted for none

Model 1 was adjusted for sex, WC, SBP, DBP

Model 2 was adjusted for sex, WC, SBP, DBP, current smoking, current drinking, education level, physical activity, Hcy, TC, LDL-C, AST, ALT, TBiL, DBiL, Albumin, eGFR, stroke, and antihypertensive drugs.

Model 3 was adjusted for age, WC, BMI, SBP, DBP, current smoking, current drinking, education level, physical activity, Hcy, TC, LDL-C, AST, ALT, TBiL, DBiL, Albumin, eGFR, stroke, and antihypertensive drugs.

**Table S3 Association between AIP and T2DM in different models stratified by sex**

| AIP Index |  | T2DM, OR (95%CI) | |  |  |
| --- | --- | --- | --- | --- | --- |
|  | Events, n (%) | Crude Model | Model 1 | Model 2 | Model 3 |
| Male |  |  |  |  |  |
| Per 1 unit increment | 510 (12.71) | 5.03 (3.69, 6.86) | 4.02 (2.86, 5.66) | 4.89 (3.10, 7.72) | 4.74 (3.00, 7.50) |
| Quartiles |  |  |  |  |  |
| Q1 (< -0.28) | 85 (8.47) | Reference |  | Reference | Reference |
| Q2 (-0.28 to < -0.10) | 102 (10.17) | 1.22 (0.90, 1.65) | 1.13 (0.83, 1.53) | 1.12 (0.78, 1.60) | 1.12 (0.78, 1.60) |
| Q3（-0.10 to < 0.10） | 108 (10.78) | 1.30 (0.97, 1.76) | 1.11 (0.82, 1.51) | 1.11 (0.76, 1.62) | 1.09 (0.75, 1.59) |
| Q4（≥ 0.10） | 215 (21.39) | 2.94 (2.25, 3.84) | 2.31 (1.73, 3.09) | 2.44 (1.66, 3.58) | 2.38 (1.62, 3.50) |
| P for trend |  | < 0.001 | < 0.001 | < 0.001 | < 0.001 |
| Female |  |  |  |  |  |
| Per 1 unit increment | 774 (18.23) | 3.34 (2.56, 4.37) | 2.93 (2.21, 3.88) | 3.20 (2.25, 4.54) | 3.20 (2.26, 4.55) |
| Quartiles |  |  |  |  |  |
| Q1 (< -0.19) | 140 (13.20) | Reference | Reference | Reference | Reference |
| Q2 (-0.19 to < -0.02) | 162 (15.27) | 1.19 (0.93, 1.51) | 1.10 (0.86, 1.41) | 1.15 (0.85, 1.56) | 1.15 (0.85, 1.56) |
| Q3（-0.02 to < 0.17） | 186 (17.56) | 1.40 (1.10, 1.78) | 1.24 (0.97, 1.58) | 1.20 (0.88, 1.64) | 1.20 (0.88, 1.64) |
| Q4（≥ 0.17） | 286 (26.88) | 2.42 (1.93, 3.02) | 2.11 (1.68, 2.66) | 2.21 (1.64, 2.99) | 2.21 (1.64, 2.99) |
| P for trend |  | < 0.001 | < 0.001 | < 0.001 | < 0.001 |
| P value for interaction |  | 0.051 | 0.092 | 0.813 | 0.821 |

Crude Model was adjusted for none

Model 1 was adjusted for age, WC, SBP, DBP

Model 2 was adjusted for age, WC, SBP, DBP, current smoking, current drinking, education level, physical activity, Hcy, TC, LDL-C, AST, ALT, TBiL, DBiL, Albumin, eGFR, stroke, and antihypertensive drugs.

Model 3 was adjusted for age, WC, BMI, SBP, DBP, current smoking, current drinking, education level, physical activity, Hcy, TC, LDL-C, AST, ALT, TBiL, DBiL, Albumin, eGFR, stroke, and antihypertensive drugs.

**Table S4 Baseline characteristics of the study population according to sex**

| Variables | Total = 8,258 | Male = 4,013 | Female = 4,245 | P-value |
| --- | --- | --- | --- | --- |
| Age, years | 64.89 ± 8.97 | 65.29 ± 9.05 | 64.51 ± 8.87 | < 0.001 |
| WC, cm | 80.45 ± 6.88 | 81.12 ± 6.94 | 79.82 ± 6.77 | < 0.001 |
| Education, n (%) |  |  |  | < 0.001 |
| < High school | 7,701 (93.26) | 3,563 (88.79) | 4,158 (97.95) |  |
| ≥ High school | 557 (6.74) | 450 (11.21) | 87 (2.05) |  |
| Current smoking, n (%) | 2,285 (27.67) | 2,029 (50.56) | 256 (6.03) | < 0.001 |
| Current drinking, n (%) | 1,860 (22.52) | 1,643 (40.95) | 217 (5.11) | < 0.001 |
| Physical activity, n (%) |  |  |  | 0.312 |
| Low | 4,540 (54.98) | 2,176 (54.22) | 2,367 (55.75) |  |
| Moderate | 1,962 (23.76) | 984 (24.52) | 977 (23.01) |  |
| high | 1,756 (21.26) | 853 (21.26) | 901 (21.24) |  |
| SBP, mmHg | 148.72 ± 17.87 | 146.72 ± 18.11 | 150.62 ± 17.43 | < 0.001 |
| DBP, mmHg | 88.40 ± 10.47 | 89.37 ± 10.57 | 87.48 ± 10.29 | < 0.001 |
| Serum homocysteine, μmol/L | 18.17 ± 10.98 | 20.71 ± 13.53 | 15.78 ± 7.06 | < 0.001 |
| Serum uric acid, μmol/L | 411.71 ± 118.50 | 458.37 ± 116.23 | 367.60 ± 102.69 | < 0.001 |
| Serum AST, U/L | 26.06 ± 11.52 | 27.00 ± 12.96 | 25.18 ± 9.89 | < 0.001 |
| Serum ALT, U/L | 18.42 ± 12.60 | 19.55 ± 13.76 | 17.35 ± 11.29 | < 0.001 |
| TBiL, μmol/L | 14.47 ± 7.10 | 15.51 ± 8.13 | 13.49 ± 5.81 | < 0.001 |
| DBiL, μmol/L | 5.52 ± 2.84 | 6.02 ± 3.42 | 5.05 ± 2.04 | < 0.001 |
| Albumin, g/L | 46.54 ± 4.08 | 46.15 ± 4.05 | 46.91 ± 4.07 | < 0.001 |
| Total cholesterol, mmol/L | 5.16 ± 1.09 | 4.95 ± 1.04 | 5.36 ± 1.10 | < 0.001 |
| Triglyceride, mmol/L | 1.66 ± 1.13 | 1.48 ± 1.06 | 1.82 ± 1.17 | < 0.001 |
| LDL-C, mmol/L | 2.95 ± 0.79 | 2.81 ± 0.75 | 3.08 ± 0.80 | < 0.001 |
| HDL-C, mmol/L | 1.61 ± 0.44 | 1.59 ± 0.45 | 1.63 ± 0.42 | < 0.001 |
| AIP | -0.04 ± 0.29 | -0.08 ± 0.29 | -0.00 ± 0.28 | < 0.001 |
| eGFR (ml/min per 1.73 m^2^) | 87.52 ± 19.87 | 85.09 ± 20.05 | 89.82 ± 19.43 | < 0.001 |
| Self-reported stroke, n (%) | 530 (6.42%) | 336 (8.37%) | 194 (4.57%) | < 0.001 |
| Self-reported diabetes, n (%) | 1,284 (15.55%) | 510 (12.71%) | 774 (18.23%) | < 0.001 |
| Antihypertensive drugs, n (%) | 5,228 (63.31%) | 2,531 (63.07%) | 2,698 (63.55%) | 0.652 |

Data are presented as the mean ± SD, or number (percentage)

Abbreviations: WC waist circumference, SBP systolic blood pressure, DBP diastolic blood pressure, AST aspartate aminotransferase, ALT alanine aminotransferase, TBiL total bilirubin, DBiL direct bilirubin, eGFR estimated glomerular filtration rate, LDL-C Low-density lipoprotein cholesterol, HDL-C High-density lipoprotein cholesterol, AIP atherogenic index of plasm.





**Fig S1. Flow chart of study participants**
